# Simple, Universal Rules Predict Trophic Interaction Strengths

**DOI:** 10.1111/ele.70126

**Published:** 2025-04-30

**Authors:** Kyle E. Coblentz, Mark Novak, John P. DeLong

**Affiliations:** ^1^ School of Biological Sciences University of Nebraska‐Lincoln Lincoln Nebraska USA; ^2^ Department of Integrative Biology Oregon State University Corvallis Oregon USA

**Keywords:** allometric scaling, consumer–resource interactions, evolution, feeding rates, macroecology

## Abstract

Many drivers of ecological systems exhibit regular scaling relationships, yet the mechanisms explaining these relationships are often unknown. Trophic interaction strengths are no exception, exhibiting scaling relationships with predator and prey traits that lack evolutionary explanations. We propose two rules to explain the scaling of trophic interaction strengths through the relationship between a predator's feeding rate and its prey's density—the so‐called predator functional response. First, functional responses allow predators to meet their energetic demands when prey are rare. Second, functional responses approach their maxima near the highest prey densities predators experience. We show that equations derived from these rules predict functional response parameters across over 2100 functional response experiments and make additional predictions such as their allometric scaling. The two rules thereby offer a potential ultimate explanation for the determinants of trophic interaction strengths, revealing ecologically realised constraints to the complex, adaptive nature of functional response evolution.

## Graphical abstract

Two simple, universal rules derived from energetic arguments predict the parameters of predator functional responses that underlie the strengths of trophic interactions. Furthermore, theory developed from these rules predicts the allometric scaling of functional response parameters, consistent patterns of predator feeding rate saturation among predators and a slow–fast continuum in predator functional responses. The two rules offer a potential ultimate explanation for the determinants of trophic interaction strengths.

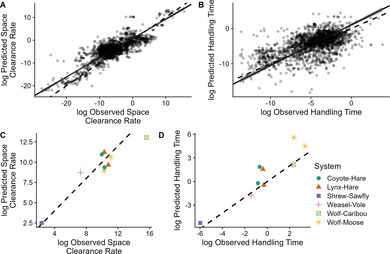

## 1 Introduction

Understanding the determinants of predator–prey interaction strengths is key to understanding the stability, diversity and dynamics of ecosystems (de Ruiter et al. 1995; McCann et al. 1998; Paine 1992; Yodzis 1981). A fundamental component of trophic interaction strengths is the predator's functional response that describes how predator feeding rates change with prey density (Holling 1959; Solomon 1949). Thousands of experiments have measured functional responses for taxa ranging from microbes to large carnivores from nearly every biome on earth (Uiterwaal et al. 2022) uncovering a number of variables influencing the underlying parameters (Coblentz et al. 2023; DeLong 2021; Pawar et al. 2012; Uiterwaal and DeLong 2020; Vucic‐Pestic et al. 2010). Principal among these variables are predator and prey body sizes, whose allometric relationships with functional response parameters serve as the basis for most dynamical food web models (Brose et al. 2006; Otto et al. 2007; Yodzis and Innes 1992). However, despite the existence and utility of such statistical relationships between functional response parameters and many traits, it remains unclear why these relationships exist as they do.

Early theory suggested that predator biomass consumption should scale to the ¾ power because organisms' metabolic rates scale to approximately the ¾ power with their masses (Yodzis and Innes 1992). More recent theory considering proximate mechanisms for the strengths of trophic interactions, such as the dimensionality of predator–prey interactions and the allometric scaling of predator and prey movement velocities, has also seen success in predicting certain functional response parameters (i.e., the space clearance/attack/search rate) and their scaling relationships (Pawar et al. 2012; Portalier et al. 2022). But other parameters (i.e., the asymptotic/maximum feeding rate or its reciprocal, handling time) remain poorly predicted, such that an understanding of the evolutionary drivers of functional responses remains missing. An encompassing means to predict predator foraging rates through theory reflecting their ultimate causes is therefore of significant interest.

We aim to provide such a theory derived from two simple rules for predator foraging. We also examine the ability of our theory to predict functional response parameters and assess several additional theoretical predictions.

## 2 Two Rules for Functional Responses

We posit that predator functional responses meet two conditions. First, a predator's feeding rate meets its energetic demands at the low prey densities it is likely to experience. This must be true for the long‐term persistence of the predator population. Although cases exist in which predator mortality or biomass loss exceeds energy intake and reproduction, as in the declining phases of predator–prey cycles, predator energy intake must balance energetic demand over the long term to avoid extirpation (McCann 2011). Second, the prey densities at which a predator's feeding rate saturates (i.e., approaches its maximum) should be near the highest prey densities it is likely to experience. This is because, first, feeding rates must saturate at some prey density (Holling 1959; Jeschke et al. 2004). Second, if feeding rates saturated at a lower prey density, predators would pay an opportunity cost for missing out on higher feeding rates at the high prey densities they experience. Third, lower handling times leading to higher, less saturated feeding rates at high prey densities: (i) have diminishing fitness returns since the highest prey densities are rarely experienced, (ii) imply less energy extracted per prey consumed (Okuyama 2010) and (iii) require larger ingestive or digestive capacities that are energetically costly (Armstrong and Schindler 2011; McWilliams and Karasov 2001; Secor et al. 1994). Although predators may rarely experience these high prey densities, selection on predators may still be strong because taking advantage of these periods of high prey abundance can have outsized effects on predator fitness. For example, Armstrong and Schindler (2011) showed that many fishes have excess digestive capacities to achieve feeding rates up to three times their average rate despite the high cost of maintaining that capacity.

Past studies on functional response evolution have largely focused on relatively simple optimization of predator feeding rates (Abrams 1982; Amarasekare 2022; DeLong and Coblentz 2022). The two rules we posit take a different view in which functional responses are the outcome of the long‐term evolution of the traits determining functional responses that balance a multitude of selective pressures. The outcome of this evolutionary process is that predators are minimally capable of persisting when prey are scarce while also taking advantage of occasionally abundant prey.

We derive two equations encapsulating these constraints. The first rule that predators must satisfy their energetic demand at low prey densities can be formalised as

(1) $$D=f_{\mathrm{low}}EM_{N}$$

where *D* is predator energetic demand in kJ/day. The rate at which prey are consumed when rare is given by its feeding rate *f*_low_, which should be well approximated by a linear functional response at low prey densities (i.e., $f_{\mathrm{low}}=aN_{\mathrm{low}}$, where a is the space clearance rate and *N*_low_ the low prey density) (Coblentz et al. 2023; Novak et al. 2024). *E* is the energy density of the prey in kJ/g, and $M_{N}$ is the mass of the prey in grams to convert the number of prey eaten to the mass of prey eaten. Although we do not explicitly account for lost or otherwise unassimilated prey energy due to undigestible parts, faeces, urine, etc. (Yodzis and Innes 1992), these processes could easily be included in Equation (1) by adjusting the prey energy density or mass.

The second rule that a predator's feeding rate should saturate near the highest prey densities it experiences can be formalised as

(2) $$I_{S}=f_{\text{high}}h$$

where $I_{S}$ is the degree of saturation (ranging between 0 and 1) and *h* is the handling time (i.e., the reciprocal of the maximum feeding rate) (Coblentz et al. 2023). Assuming a Holling Type II functional response, $f_{\text{high}}=\frac{aN_{\text{high}}}{1+\mathrm{ah}N_{\text{high}}}$, where $N_{\text{high}}$ is the high density of prey likely to be experienced by the predator. $I_{S}$ also may be interpreted as the fraction of time a predator individual is busy handling prey, or as the fraction of individuals in a predator population handling prey at any given point in time assuming a steady‐state (Supporiting Information) (Coblentz et al. 2021; Lafferty et al. 2015; Novak et al. 2017). We focus on the Holling Type II version of the functional response over the Michaelis–Menten form (i.e., $f=\frac{f_{\max}N}{c+N}$ where f is the feeding rate, $f_{\max}$ is the maximum feeding rate and c is the half‐saturation constant or the prey density at which $f=\frac{1}{2}f_{\max}$). We do so for two reasons. First, the Holling Type II functional response parameters have clear mechanistic interpretations: a is the space that would be cleared by a predator in the absence of a time cost to handling prey, and h is the time taken away from searching for prey once a prey is captured. Second, the parameters of the Holling Type II functional response have practical interpretations related to feeding rates at high and low prey densities: a is the slope of the functional response at the origin, and $\frac{1}{h}$ is the maximum feeding rate.

Given Equations (1) and (2), we solve for the space clearance rate and handling time as

(3) $$a=\frac{D}{N_{\mathrm{low}}EM_{N}}$$

and

(4) $$h=\frac{I_{S}N_{\mathrm{low}}EM_{N}}{DN_{\text{high}}\left(1-I_{S}\right)}$$

### 2.1 Auxiliary Predictions

Beyond providing equations for the space clearance rate and handling time parameters, Equations (1) and (2) also make auxiliary predictions. Here, we focus on three of these related to (i) the degree of feeding rate saturation across prey densities experienced by predators, (ii) the relationship between space clearance rates and handling times and (iii) the allometric scaling of space clearance rates and handling times.

(i) Regarding the saturation of predator feeding rates with prey densities, we assume that the functional response is unsaturated at low prey densities and approaches saturation at the high prey densities experienced by predators (Equations 1 and 2). Given these two assumptions, we also can assess the prey density at which the predator's feeding rate is half saturated. This occurs when the prey density is equal to $\frac{1}{\mathrm{ah}}$ (the half saturation constant in the Michalis‐Menten formulation). Using Equations (3) and (4), we solve for the half saturation constant as

(5) $$\frac{1}{\mathrm{ah}}=\frac{\left(1-I_{S}\right)N_{\text{high}}}{I_{S}}$$

Thus, our theory predicts that, despite different predators experiencing different ranges of prey densities, their degree of saturation across those density ranges is invariant.

(ii) Regarding the relationship between space clearance rates and handling times, it follows from Equation (5) that a and h are related as

(6) $$a=\frac{I_{S}}{\left(1-I_{S}\right)N_{\text{high}}h}$$

and taking the natural log of both sides,

(7) $$\ln\left(a\right)=\ln\left(\frac{I_{S}}{\left(1-I_{S}\right)N_{\text{high}}}\right)-\ln\left(h\right)$$

Our theory therefore predicts an inverse (or negative log–log‐linear) relationship between space clearance rates and handling times among predators feeding on prey with similar high population sizes.

(iii) Last, several of the parameters on the right‐hand sides of Equations (3) and (4) have well‐known scaling relationships with species' body masses. Specifically, energetic demand defined as predator metabolic rate scales with predator mass (Kleiber's Law; Brown et al. 2004; Kleiber 1932) and prey densities scale with prey mass (Damuth's Law; Damuth 1981). As we can treat the remaining parameters as unrelated to body size, this suggests that the theory outlined here should be capable of predicting the allometric scaling of a and h.

Below, we take advantage of the FoRAGE database of functional response experiments to examine the predictions of our theory (Uiterwaal et al. 2022). We first combine FoRAGE with databases on mass‐abundance and metabolic scaling relationships to predict functional response parameters across most studies in FoRAGE. Then, for a subset of FoRAGE studies performed in the field, we examine our theory's ability to make predictions using system‐specific information. Last, we examine the evidence for the auxiliary predictions made by our theory.

## 3 Methods

### 3.1 Databases, Data Handling and Predictions

We evaluate the ability of our theory to predict functional response parameters by comparing measured functional response parameters to predictions derived using estimates of the parameters on the right‐hand sides of the equations. To do so, we bring together two databases: the FoRAGE compilation from Uiterwaal et al. (2022) and a data compilation of eukaryotic species' masses, metabolic rates, and population densities from Hatton et al. (2019). FoRAGE contains functional response parameter estimates from 3013 functional response experiments along with additional information, including predator and prey body masses, for most studies. Thus, FoRAGE provides the measured space clearance rates and handling times and values for prey mass in wet weight ($M_{N}$). To predict space clearance rates and handling times using Equations (3) and (4), we still require estimates of energetic demand (D), prey energy density (E), high and low prey densities ($N_{\mathrm{low}}$ and $N_{\text{high}}$) and the degree of feeding rate saturation at high prey densities ($I_{S}$).

To obtain estimates of low and high prey densities likely to be experienced by predators (*N*_low_ and *N*_high_), and predator energy demand (*D*), we combined FoRAGE with mass scaling data from Hatton et al. (2019). To estimate $N_{\mathrm{low}}$ and $N_{\text{high}}$, we used the data from Hatton et al. (2019) containing species' masses and their measured densities in aerial m^2^. These density estimates include density estimates from the same species in different places or times, as well as estimates across different species. We assume that the variation in the density data reflects both variation in prey densities a predator might experience over space and time and among rare and common species with similar masses. To estimate $N_{\mathrm{low}}$ and $N_{\text{high}}$ for the prey in FoRAGE, we performed Bayesian log–log regressions of density on mass separately for prokaryotes (*n* = 635), protists (*n* = 301), invertebrates (*n* = 778), ectotherm vertebrates (*n* = 404), mammals (*n* = 2852) and birds (*n* = 603). Using the prey classifications and masses in FoRAGE, we then estimated the posterior predictive distribution for each study‐specific prey and extracted the 10th and 90th percentiles as estimates of *N*_low_ and *N*_high_, respectively. To estimate *D*, we performed Bayesian log–log regressions of basal metabolic rate in kJ/d on predator mass in g separately for protists (*n* = 365), invertebrates (*n* = 4559), ectotherm vertebrates (*n* = 616), mammals (*n* = 1059), and birds (*n* = 492). We then used the median predicted metabolic rate of each predator in FoRAGE based on its classification and mass as our estimate of *D*. For the Bayesian regressions, we used flat priors on the intercept and slope, and a Student‐t prior with degrees of freedom = 3, location = 0, and scale = 2.5 on the standard deviation. We approximated the posterior distribution using 1000 samples each from 4 Markov chains after a 1000 sample warmup. We performed the regressions using the R package ‘brms’ (Bürkner 2017) using Stan (Stan Development Team 2024) through the backend cmdstanr (Gabry et al. 2024).

With estimates of $N_{\mathrm{low}}$, $N_{\text{high}}$, and D, we then needed estimates of E and $I_{S}$. We assume that these values are approximately constant, setting E = 5.6 kJ/g wet weight following Brown et al. (2018) and $I_{S}=0.9$ (i.e., feeding rates saturated at 90% of their maximum). Our results are not sensitive to reasonable choices of values for $I_{S}$ or for the percentiles used for $N_{\mathrm{low}}$ and $N_{\text{high}}$ (see Supporting Information).

Although the full FoRAGE database contains 3013 experiments, we restricted our predictions to:

1. Studies include living prey that are not eggs. We do not include studies using eggs as prey as it is unclear whether mass scaling relationships apply to eggs.
2. Studies with handling time estimates greater than 1 × 10^−6^ days. We did so following previous studies suggesting that studies within FoRAGE with lower values than this cutoff are those for which Type II functional responses have poor fits (Coblentz et al. 2023; Kiørboe and Thomas 2020; Uiterwaal and DeLong 2020).
3. Studies with both predator and prey masses. We excluded studies without this information because it is what allows a link between the FoRAGE database and empirical mass scaling relationships.

After excluding the studies that did not meet these criteria, we were left with 2162 functional response measurements.

Combining the estimates of *N*_low_, *N*_high_, *D*, *E*, and *I*_S_, we predicted the space clearance rates and handling times for each predator–prey pair in the reduced version of FoRAGE. We compared these predictions to observed values using reduced major axis regression with the R package ‘lmodel2’ (Legendre 2018).

### 3.2 Field‐Study Specific Predictions

Forty of the 2162 FoRAGE experiments in our reduced dataset were performed in the field. Because these field studies include high and low abundances of prey observed during the studies, they provide an opportunity to test functional response parameter predictions using system‐specific information rather than mass scaling relationships. To avoid circularity, we excluded studies that estimated predator kill rates using the proportion of predator diets consisting of a prey type coupled with the average mass of that prey and the predator's daily energetic demand to estimate the number of prey consumed per day. For the remaining studies, we performed a literature search to find information on predator energetic demand (*D*) and prey energy density (*E*). For energy demand, we used estimates of daily energy expenditure, if available. Otherwise, we used basal metabolic rate. For energy density, we used species‐specific estimates of kJ/g if they were available. Otherwise, we used studies of prey body composition that gave the percent of prey body mass composed of protein and fat. We used conversions of 16.74 kJ/g for protein and 33.47 kJ/g for fat to calculate prey energy density (Chizzolini et al. 1999; Menzies et al. 2022). We were able to find the requisite information for seven studies of six mammalian predator–prey pairs. For these studies, we assumed the highest and lowest recorded prey densities as estimates of *N*_low_ and *N*_high_. Details and references for the estimates are given in the Supporting Information. As the estimate of mass, we used the mass in FoRAGE (Uiterwaal et al. 2022). FoRAGE presents the mass reported in the original study where possible, but if the mass was not available from the original study, the authors of FoRAGE took a stepwise process to estimate masses, for example, using length–mass relationships or data from sources other than the original study [for details see Uiterwaal et al. (2022)].

### 3.3 Auxiliary Predictions

- To examine whether our theory's predicted relationship between the predator's half‐saturation constant and prey densities held, we used the values of the space clearance rates and handling times from FoRAGE and the mass‐abundance scaling prediction for high prey abundances. To assess the accuracy of the predictions, we calculated the correlation and performed a major axis regression on the observed versus predicted half‐saturation constants.
- To examine our theory's prediction of the relationship between space clearance rates and handling times given *I*_S_ and the high density of the prey, we used the values of the space clearance rates and handling times from FoRAGE and the mass–abundance scaling predictions of the high prey abundances. To assess the accuracy of the prediction of the relationship between space clearance rates and handling times, we calculated the correlation and performed a major axis regression on the observed versus predicted space clearance rates.
- To assess whether our theory could predict the allometric scaling of space clearance rates and handling times, we derived equations for the scaling of space clearance rates and handling times, assuming that predator energy demand (*D*) has a power‐law scaling with predator mass, that low and high prey densities (*N*_low_ and *N*_high_) scale with prey mass, and that predator and prey masses scale with one another. We assumed the other variables are constants unrelated to predator or prey mass. Derivations of these equations are given in the Supporting Information. We then used log–log least squares regressions to test our predictions, obtaining estimates of the empirical relationships within FoRAGE across the different classifications used in the mass‐abundance and mass‐metabolism regressions between prey masses and abundances, predator masses and energy demand, and predator and prey masses (see Section 3.1).

All analyses were performed in R using v. 4.3.1 (R Core Team 2023).

## 4 Results

### 4.1 Prediction of *a* and *h*

**FIGURE 1:**
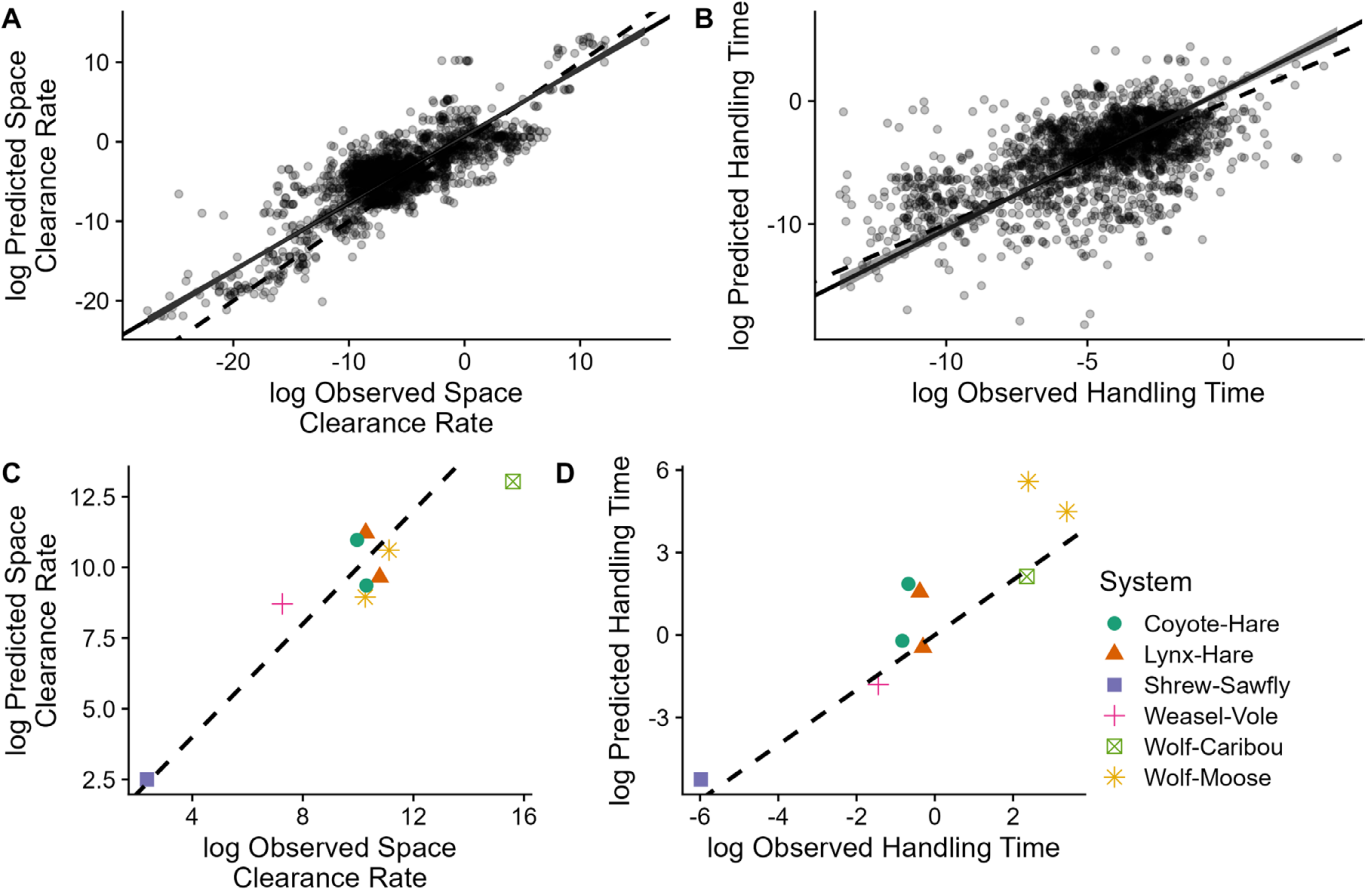
Two simple rules derived from predator feeding rates at low and high prey densities predict space clearance rates and handling times using either generic estimates from empirical mass scaling relationships (A, B) or system‐specific estimates from field studies (C, D). Dashed lines are 1:1 lines, solid lines are major axis regressions, and the grey ribbons around the solid lines in A and B are 95% confidence intervals. See the Supporting Information for versions of panels A and B with colour‐coded predator and prey body size information.

The two rules performed well in predicting empirically estimated space clearance rate and handling time values across their respective 19 and 8 orders of magnitude in variation (Figure 1; log space clearance rate correlation coefficient = 0.82, *R* ^2^ of 1:1 line = 0.81; log handling time correlation coefficient = 0.57, *R* ^2^ of 1:1 line = 0.75). The major axis regression slopes between observed and predicted space clearance rates (*ß* = 0.85, 95% Confidence Interval [CI] = 0.82, 0.87) and handling times (*ß* = 1.14, 95% CI = 1.08, 1.22) suggest a tendency for the slight overestimation of space clearance rates and underestimation of handling times at low values, and the underestimation of space clearance rates and overestimation of handling times at high values (Figure 1). Alternative means for determining low and high prey densities and levels of predator saturation shift the points in Figure 1, but do not influence the predictive power of the two rules (Supporting Information). Indeed, the tendency for over/under‐estimation likely reflects insightful across‐system variation not reflected in estimates from mass‐scaling relationships (see below).

We also predicted the functional response parameters for the subset of the studies that were field studies. For the seven studies of mammalian predators, we again find that the predictions fall near the 1:1 line but without the same systematic tendency for over‐/underestimation (Figure 1C,D).

### 4.2 Auxiliary Predictions

**FIGURE 2:**
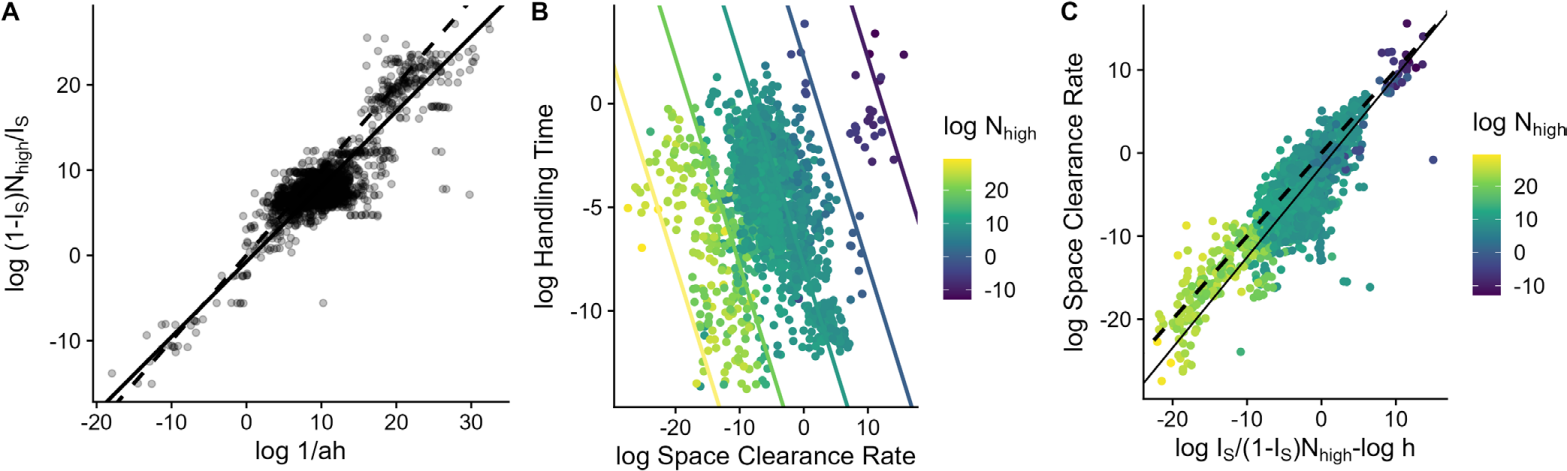
Two simple rules predict empirical estimates of the half‐saturation constant (*1/ah*) suggesting consistent patterns of feeding rate saturation among predators (A), as well as the relationship between space clearance rates and handling times (B, C) suggesting predators eating prey of a given size with higher space clearance rates have lower handling times. The dashed lines in A and C are the 1:1 lines and the solid lines are major axis regressions. The lines in B are the predicted relationships between space clearance rates and handling times for different values of *N*_high_. See the Supporting Information for versions of panels A with colour‐coded predator and prey body size information.

**FIGURE 3:**
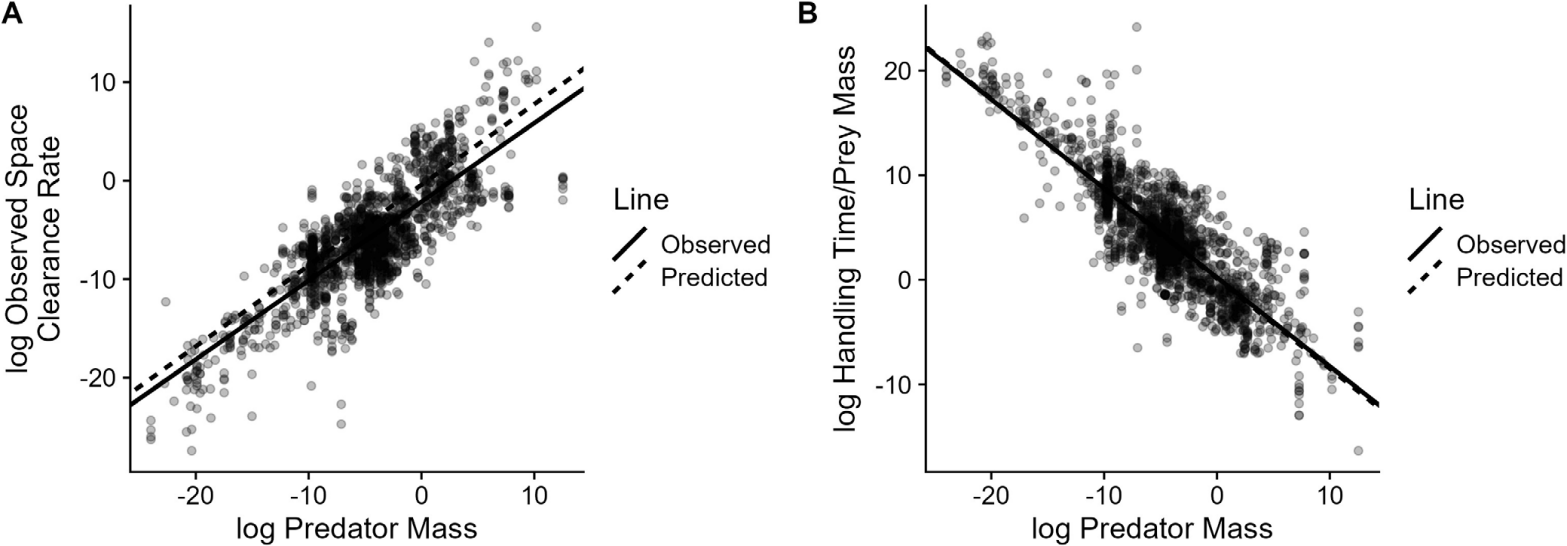
Two simple rules predict the allometric scaling of predator functional response parameters. Functional response rules and empirical scaling relationships between parameters and prey and predator masses predict that the log space clearance rate increases with log predator mass with a slope of 0.82 (observed empirical estimate: 0.8, A) and that the log of the prey mass‐specific handling time (handling time/prey mass) decreases with log consumer mass with a slope of −0.87 (observed empirical estimate: −0.86; B). See the Supporting Information for versions of the panels with colour‐coded predator and prey body size information.

- The relationship between the empirical half‐saturation constant values and high prey densities, modified by the degree of saturation, supports our theory's prediction (correlation = 0.86, slope of major axis regression [95% CI] = 0.88 (0.86, 0.9), Figure 2A; see the Supporting Information for a sensitivity analysis).
- Space clearance rates and handling times were inversely related to each other when accounting for the high prey densities (Figure 2B,C). Without accounting for the high prey densities, there is little apparent relationship between space clearance rates and handling times (Figure 2B). However, once the high prey densities are accounted for, the negative relationship between space clearance rates and handling times is evident (see the colour coding in Figure 2B). Examining the prediction of the relationship between handling times modified by high prey densities and the degree of predator feeding rate saturation at high prey densities supports our theory's prediction, albeit with a tendency to underpredict the space clearance rates (correlation = 0.84, intercept of major axis regression [95% CI] = −1.5 [−1.6, −1.4], slope of major axis regression [95% CI] = 1.07 [1.04, 1.1]).
- Space clearance rates and handling times scale with predator and prey body masses as predicted. Specifically, the empirical scaling of the parameters we consider as being related to body size (those besides $I_{S}$ and E) are $N_{\mathrm{low}}\propto M_{N}^{-0.94}$, $N_{\text{high}}\propto M_{N}^{-0.9}$, and $D\propto M_{P}^{0.87}$ where *M*_P_ is the mass of the predator and the other parameters are defined above. Given these scaling relationships and the first rule, it follows that the allometric relationship for the space clearance rate should be $a\propto M_{P}^{0.87}M_{N}^{-0.06}$. Given that in the dataset $M_{N}\propto M_{P}^{0.79}$, we therefore predict that $a\propto M_{P}^{0.82}$. This prediction matches the observed scaling of $a\propto M_{P}^{0.8}$ (95% CI = 0.78, 0.83; Figure 3A). Similarly, the allometric relationship for handling time should be $h\propto M_{N}^{0.96}M_{P}^{-0.87}$. Thus, we predict $\frac{h}{M_{N}}\propto M_{P}^{-0.87}$. This prediction also matches the observed scaling of $\frac{h}{M_{N}}\propto M_{P}^{-0.85}$ (95% CI = −0.87, −0.82; Figure 3B; See Supporting Information for derivations of scaling relationships).

## 5 Discussion

We demonstrate that two simple rules predict the parameters of predator functional responses and their scaling relationships. In contrast to prior work, our theory provides an ultimate explanation for the strengths of trophic interactions rather than focusing on proximate mechanisms (e.g., predator and prey encounter rates, capture successes, etc.). Our approach generates predictions of space clearance rates of predators with similar accuracy as prior theory (i.e., most predictions within ~3 orders of magnitude of the observed values; Pawar et al. 2012; Portalier et al. 2022), but also predicts handling times, which have been predicted poorly by previous work (Portalier et al. 2022). In addition, our theory offers two practical advantages. First, it uses parameters that are generally easier to measure than those required by previous theory (e.g., predator movement velocities and prey detection distances). Second, our theory's foundation in ultimate causes suggests potential applications beyond environmental conditions and taxa in which trophic interaction strengths have been measured, addressing a limitation of current statistical approaches to prediction (DiFiore and Stier 2023).

Our two rules emerge from a view of functional responses as products of a long‐term eco‐co‐evolutionary process involving predators, their prey, and the environment including the effects of other species and their interactions (DeLong 2020; Gutiérrez Al‐Khudhairy and Rossberg 2022; Wickman et al. 2024). A predator's fitness depends not only on its ability to procure food; it also depends on its ability to avoid its own predators, find mates, etc. (Jeschke 2007). Therefore, we suggest that eco‐evolutionary processes combine such that trophic interaction strengths reflect the ability of a predator to minimally meet energetic demands during busts of low prey densities while also taking advantage of instances of booms of high prey densities. This contrasts with previous work focusing on the optimisation of space clearance rates and handling times to maximise energy intake. This view also may help explain why consumers tend not to overexploit their prey (Gutiérrez Al‐Khudhairy and Rossberg 2022; Vuorinen et al. 2021). As many complex behaviours and phenotypes are the product of multiple traits under multiple selective pressures, our approach of focusing on functional outcomes rather than specific processes may offer a way forward for understanding and predicting other complex phenotypes such as thermal responses and competitive abilities and their resulting ecological scaling relationships.

The theory developed here also makes predictions beyond the values of space clearance rates and handling times. The first is the prediction that predators show consistent patterns in how their feeding rates saturate across prey densities: feeding rates are largely unsaturated at low prey densities, reach half‐saturation at an intermediate fraction of the highest prey densities, and only become fully saturated at the highest prey densities encountered. This result is consistent with that of Coblentz et al. (2023) who found that predator feeding rates were generally unsaturated at the typical prey densities predators are likely to experience. Indeed, given the highly skewed frequencies of species abundances spatially and temporally in nature (Brown et al. 1995; Halley and Inchausti 2002), the predicted prey density at which half‐saturation occurs within our data is typically 2.4 times higher (standard deviation = 0.4) than the predicted median prey density. This suggests that predators only rarely feed near their maximum feeding rates, implying that predation pressure should dynamically change with changes in prey densities.

The second auxiliary prediction from our theory is a negative relationship between space clearance rates and handling times, a pattern previously found by Kiørboe and Thomas (2020). These authors interpreted this relationship as a slow–fast continuum in trophic interactions that appeared to contradict the gleaner‐opportunist tradeoff—a fundamental ecological concept that helps explain species coexistence under variable resources (Armstrong and McGehee 1980; Grover 1990). Under the gleaner‐opportunist tradeoff, ‘gleaners’ perform better at low resource levels while ‘opportunists’ take advantage of high resource situations. If ingestion rates directly determine growth rates, the gleaner‐opportunist tradeoff would predict a positive relationship between space clearance rates and handling times (Kiørboe and Thomas 2020), a pattern that is opposite to that which we and Kiørboe and Thomas (2020) observed. Our results provide a potential resolution to this apparent contradiction by considering predator energy demand. Our theory suggests that the negative relationship between space clearance rates and handling times for predators consuming similarly sized prey occurs due to differences in energy demand—energy demand appears in the numerator and denominator of the equations for space clearance rates and handling times, respectively. Thus, predators with higher energy demands exhibit higher space clearance rates and lower handling times, whereas predators with lower energy demands exhibit the opposite pattern. Although high‐energy‐demand predators may consume more prey at low prey availability, they also incur higher energetic costs under these conditions compared to low‐energy‐demand predators. This energetic tradeoff provides a mechanism for the co‐occurrence of a gleaner‐opportunist tradeoff with the presence of a slow‐fast continuum in functional responses parameter.

The third auxiliary prediction from our theory is the allometric scaling of space clearance rates and handling times that play a central role in dynamical food web models (Brose et al. 2006; Otto et al. 2007; Yodzis and Innes 1992). Existing allometric scaling relationships of functional response parameters generally fall into two groups. The first are studies following Yodzis and Innes (1992) who argued that predators' maximum feeding rates should scale with predator mass to the same exponent as metabolic rate and that the half‐saturation constant in units of prey mass is independent of prey and predator masses. Translating this scaling to our own, we find that the two approaches predict similar allometric scaling estimates (prey‐mass specific scalings of handling times of −0.87 under both approaches and space clearance rate scalings of 0.82 in our theory and 0.87 for Yodzis and Innes (1992); Supporting Information S6). Although this convergence in predictions is likely due to the central role played by predator metabolic rate scaling, we note that our approach also predicts the pre‐factor of the relationship (Figure 3) and provides a potential explanation for the lack of allometric scaling of the half‐saturation constant that is left unexplained in Yodzis and Innes (1992). The second group of allometric scaling relationships focuses on the space clearance rate (McGill and Mittelbach 2006; Pawar et al. 2012; Rall et al. 2012) and derives different scaling relationships between space clearance rates and predator mass depending on whether predators and prey interact in two or three dimensions. These studies predict that space clearance rates scale with predator body mass to an exponent between 0.58–0.68 in two dimensions and 0.92–1.07 in three dimensions. Our predicted and observed scaling exponent of 0.82 falls between these ranges. Unfortunately, we cannot derive dimension‐specific allometric scaling relationships using our current data because the dataset from which we derive our abundance estimates (Hatton et al. 2019) is in a single dimension (aerial m^2^) and does not have information on the dimension in which organisms live or interact with their predators. Future work incorporating dimension‐specific scaling relationships into our theory will thus be useful for integrating the ultimate and proximate mechanisms determining trophic interaction strengths.

The rules we derive make testable predictions regarding how the environment and global climate change are likely to influence trophic interaction strengths, as many of the parameters describing our rules are sensitive to the environment. For ectotherms, predator energetic demand is expected to increase with increasing temperature (Brown et al. 2004), whereas prey population size, body size and potentially energy density are likely to decrease (Amarasekare and Savage 2012; Atkinson 1994; Bernhardt et al. 2018; Brett et al. 1969; Outhwaite et al. 2022). These changes will alter the functional response parameters required to satisfy our rules, suggesting the potential for maladaptive trophic interactions under climate change. Changes in parameters also occur over other environmental gradients, such as productivity gradients (Novak 2013), which may allow for the prediction of changes in top–down interaction strengths and their ecosystem consequences. Furthermore, although our analyses focused on interspecific differences in trophic interactions, this theory also may be applicable to intraspecific variation in predators and prey to explain intraspecific variation in functional response parameters (Bolnick et al. 2011; Coblentz et al. 2021).

The success of our theory in predicting functional response parameters opens several promising directions for future theoretical and empirical insights into trophic interaction strengths. First, our theory provides predictions about which trophic interactions within communities are likely to be strong, helping to identify potential keystone interactions that may disproportionately impact communities. Second, although our theory has focused on pairwise trophic interactions, it provides the foundation for a theory on generalist predators to embrace the additional complexity present in food webs in which predators consume multiple prey species. Last, although we have focused specifically on traditional predators, it may be possible to extend the theory to other consumer types such as herbivores, detritivores, and parasites. By deepening our understanding of the ultimate causes governing trophic interaction strengths, this work promises to provide important insights into the functioning and dynamics of ecosystems and their responses to ongoing global change.

## Author Contributions

K.E.C., M.N., and J.P.D. designed the research. K.E.C. developed the theory with input from M.N. and J.P.D., performed the analyses, and wrote the first draft of the manuscript. All authors contributed to revisions.

## Conflicts of Interest

The authors declare no conflicts of interest.

### Peer Review

The peer review history for this article is available at https://www.webofscience.com/api/gateway/wos/peer‐review/10.1111/ele.70126.

## Supporting information


Data S1.

## Data Availability

All data and code for analysing data are permanently archived at: https://doi.org/10.5281/zenodo.15121686.
